# Elastofibroma Dorsi: A Rare Connective Tissue Tumor

**DOI:** 10.7759/cureus.6874

**Published:** 2020-02-04

**Authors:** Mohamed S Suliman, Zachary S Higginbothom, Ahmed Amro, Colin McCorkle, Elizabeth Saunders

**Affiliations:** 1 Internal Medicine, Marshall University, Joan C. Edwards School of Medicine, Huntington, USA; 2 Cardiology, Marshall University, Joan C. Edwards School of Medicine, Huntington, USA

**Keywords:** elastofibroma, benign tumor, soft tissue mass

## Abstract

Elastofibroma dorsi (ED) is a benign connective tissue tumor that most commonly occurs on the inferior pole of the scapula. It can be found incidentally on radiologic imaging or due to clinical symptoms. Patients may become apprehensive due to it mimicking new malignancy or recurrence of prior malignancy. Treatment is only recommended in symptomatic cases and biopsy is usually unnecessary. We present a case of a 70-year-old female status-post lung cancer resection who was found to have a lump at the inferior pole of her right scapula. She was seen by multiple different specialties and subsequently, a biopsy confirmed her mass to be consistent with ED. Since ED is a benign soft tissue tumor, educating physicians is of utmost importance to avoid pursuing unnecessary diagnosis and to thereby decrease the cost of care to the patient. Therapeutic excision should only be performed in symptomatic patients and observing these lesions in asymptomatic patients would be sufficient.

## Introduction

Elastofibroma dorsi (ED) is a rare benign connective tissue tumor that is typically found on the inferior pole of the scapula [1]. First described in 1959 by Jarvi and Saxen, ED can cause apprehension due to its embedded location and large size frequently greater than 5 centimeters [2,3]. It is most prevalent in Japan, elderly females, patients with a history of manual labor involving repetitive shoulder movement, trauma, and a family history of ED. Clinical features include but are not limited to pain, swelling, stiffness, and a clunking sensation with movement; however, most patients are asymptomatic. ED typically stains cluster of differentiation (CD)34+ and vimentin positive, and is histologically composed of fat, collagen, and degenerated coarse band fibers that resemble elastic tissue [2,4]. Characteristic findings on radiologic imaging include a poorly circumscribed, solitary heterogenous soft tissue mass that can often blend in on computed tomography (CT) scan with surrounding soft tissue due to similar densities [1,5]. Ultrasonography (U/S) is the most cost-effective diagnostic tool in conjunction with a high degree of clinical suspicion. Confirmatory diagnosis involves a biopsy but is usually unnecessary. Surgical intervention is only recommended for patients with symptomatic ED [6]. In a review of cases, it has been reported that these lesions have a tendency to occur at the site of prior thoracoscopic surgeries and are known to mimic the recurrence of port site cancer [7]. We present a case of a 70-year-old female status-post lung cancer resection who was found to have a lump at the inferior pole of her right scapula.

## Case presentation

**Figure 1:**
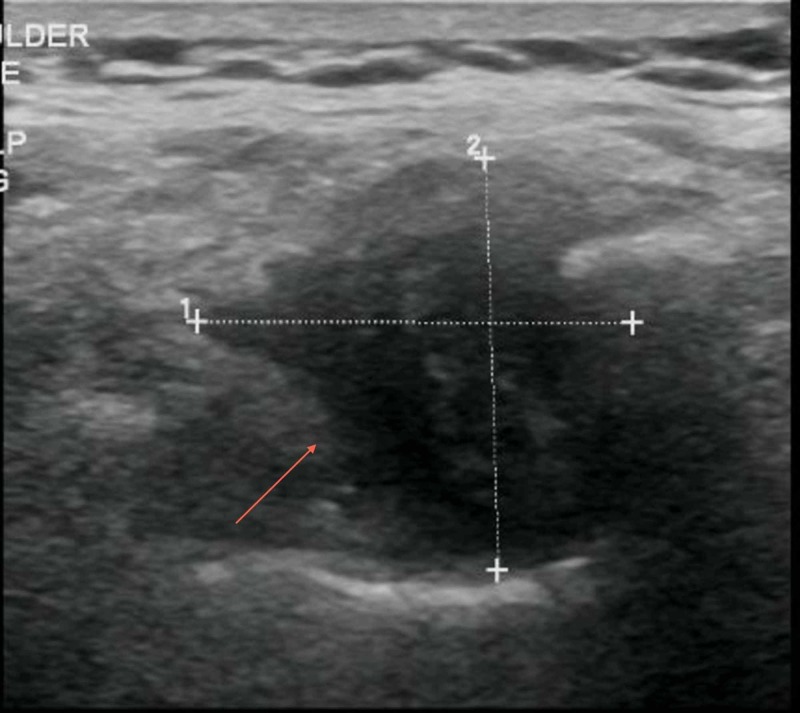
Ultrasound of right periscapular region showing a complex hypo-echoic mass

A 70-year-old Caucasian female with a past medical history significant for severe chronic obstructive pulmonary disease (COPD), lung cancer status-post resection 15 years prior, and osteoporosis presented to the urgent care in March of 2019 complaining of a lump on the right side of the upper back. This happened to be the at the site of a previous right-sided thoracotomy where she underwent segmentectomy of the right upper lobe, and a right upper lobe lobectomy in 2004. No radiation or chemotherapy was administered at the time of surgery and she had remained cancer free with no reoccurrence based on her yearly pulmonary follow ups. Upon initial presentation, she stated that she had noticed the mass over the past year and although it was painless, it had raised concern due to its progressive size. She denied fever or recent history of trauma. On physical examination, the surgical scar from the thoracotomy appeared well healed with no signs of infection. A non-tender mass was palpated just under the surgical scar and at the time of exam, measured approximately 2-3 cm in diameter but became more prominent with movement of the scapula. U/S showed a 3 cm complex mass and magnetic resonance imaging (MRI) was recommended for further evaluation (Figure 1).

**Figure 2:**
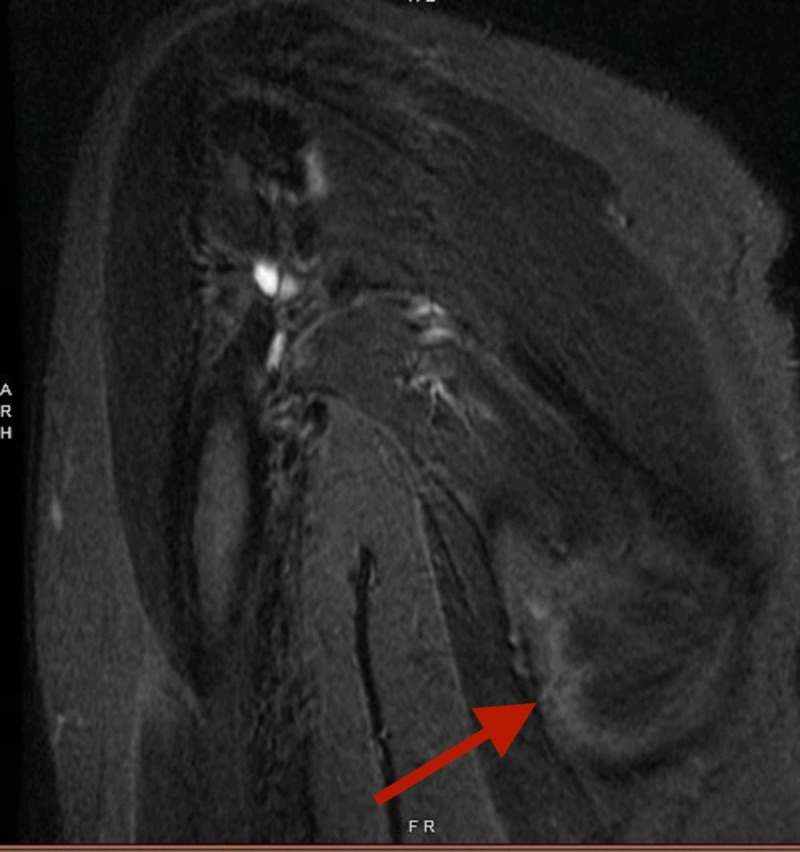
Heterogenously enhancing mass demonstrated along the right posterior lateral chest wall

Her MRI showed a heterogeneously enhancing mass along the right posterior lateral chest wall (Figure 2). The mass had increased in size compared to the chest CT scan from 2015 that was routinely performed for lung cancer follow up. Biopsy was recommended, and the patient was referred to the oncology clinic.

The patient was evaluated by oncology and at that time, the differential diagnosis included elastofibroma, desmoid tumor, sarcoma, and recurrent lung cancer. A CT-guided biopsy was performed, and the pathology report was consistent with a diagnosis of elastofibroma. The patient's oncologist recommended no surgical intervention of the mass due to its benign nature.

## Discussion

During the 12th Histopathology Scandinavian Congress in 1959, ED was first described. It was postulated that this soft tissue growth was due to degenerative and regenerative processes along the thoracic fascia. Later in 1969, Jarvi et al. published an in-depth manuscript and confirmed that scapular movements along the thoracic fascia cause friction which leads to the development of the mass. Gross histopathological features also provide sufficient reasoning for the slow growth of this mass [2,8]. In 1987 Fukuda et al. postulated that ED is formed as a result of abnormal elastofibrogenesis which undergoes degeneration due to lack of vascular support for the tissue [9,10]. Lastly, familial predisposition and chromosomal regions Xq12-q22 and 19 have also been proposed by authors for the development of ED [8,10]. Although these hypotheses present convincing theories as to the etiology of ED, the exact etiology has not been elucidated.

Women over the age of 50 have a higher occurrence of ED [8-10]. The female to male ratio has been reported to be as high as 13:1 [8,11,12]. It is asymptomatic and is usually an incidental finding on imaging. The most likely location of ED is at inferior pole of the scapula where it is attached to the chest wall beneath the serratus anterior muscle. It is usually found at a single site but has been reported to occur bilaterally in 10-66% of patients [2,8,11,13]. Other locations like the stomach, peritoneum, ischial tuberosities, mediastinum, intraspinal spaces, deltoid muscle, and foot have also been reported although less common [9]. ED is mostly asymptomatic but 50% of the patients experience symptoms of stiffness and discomfort upon movement.

Diagnostic modalities include ultrasonography, MRI, and CT. U/S can be an extremely useful modality in conjunction with an astute clinician. No further imaging is necessary if the mass has regular boarders, is not cystic and fits the classic presentation of ED: a slow-growing subscapular mass in a woman over the age of 50 with or without previous risk factors [14,15]. On U/S, ED appears as a multilayered mass with a well-defined fasciculated pattern. If the mass has a non-fasciculated pattern on U/S, then CT/MRI should be pursued to further characterize the lesion and form a diagnosis. Although this imaging algorithm has been recommended, CT or MRI are more frequently utilized as a first-line imaging modality to diagnose ED. ED on CT/MRI appears as a heterogenous, poorly defined, soft tissue mass with strands of fat. If diagnosis based on imaging is not felt to be sufficient, then a definitive diagnosis can be made by tissue biopsy [8]. As with our patient, a biopsy was pursued to definitely define the mass and once confirmed to be ED, the mass was not surgically removed. Conservative therapy and observation is the recommended approach for elderly patients who are asymptomatic as ED has never been reported to have malignant transformation. If the patient experiences pain or discomfort, marginal surgical excision would be warranted to alleviate the symptoms [7].

## Conclusions

ED is a benign soft tissue tumor which occurs most commonly in elderly female patients. Clinicians with awareness of this tumor can save time and avoid pursuing extensive work up by using an U/S and a thorough physical exam. Rarely is it found to be symptomatic and even then, it merely causes discomfort with mobilization. If asymptomatic, close observation of the mass is sufficient. 
